# Professional satisfaction in nursing during the COVID-19 pandemic *

**DOI:** 10.1590/1518-8345.6364.3894

**Published:** 2023-05-12

**Authors:** Manuela Vilas Boas Pirino, Carlito Lopes Nascimento, Ariane Polidoro Dini

**Affiliations:** 1 Universidade Estadual de Campinas, Faculdade de Enfermagem, Campinas, SP, Brasil; 2 Becaria de la Coordenação de Aperfeiçoamento de Pessoal de Nível Superior (CAPES), Brasil.; 3 Universidade Estadual de Feira de Santana, Feira de Santana, BA, Brasil.

**Keywords:** Pandemics, Occupational Health, Job Satisfaction, Nursing Administration Research, Working Conditions, Occupational Stress, Pandemias, Salud Laboral, Satisfacción en el Trabajo, Investigación en Administración de Enfermería, Condiciones de Trabajo, Estrés Laboral, Pandemias, Saúde do Trabalhador, Satisfação no Trabalho, Pesquisa em Administração de Enfermagem, Condições de Trabalho, Estresse Ocupacional

## Abstract

**Objective:**

to assess the job satisfaction of nursing professionals who worked in care and management during the COVID-19 pandemic.

**Method:**

cross-sectional epidemiological study, with the participation of 334 nursing professionals of a teaching hospital. Absolute and relative frequencies of qualitative variables and means of numerical variables were calculated. The mean scores of the domains of the Job Satisfaction Survey were compared with sociodemographic-labor variables. Student’s t-test, Mann-Whitney test, and ANOVA test were applied and followed by Tukey, Kruskal-Wallis, or Dunn’s post-test to verify the statistical significance of the results with a critical level of 0.05.

**Results:**

90 professionals were satisfied with their work, three professionals were dissatisfied and 241 professionals were ambivalent.

**Conclusion:**

ambivalence was evidenced among nursing workers regarding their job satisfaction during the period of the COVID-19 pandemic. The findings indicate a path for managers and health policymakers to invest in career plans and work environments that improve the working conditions of nursing workers.

## Abstract (es)

**Objetivo:**

evaluar la satisfacción laboral de profesionales de enfermería actuantes en atención y gestión durante la pandemia de COVID-19.

**Método:**

estudio epidemiológico de corte transversal. Participaron 334 profesionales de enfermería de un hospital de enseñanza. Fueron calculadas las frecuencias absolutas y relativas de las variables cualitativas y los promedios de las variables numéricas. Se compararon los promedios de resultados de los dominios de la escala de satisfacción profesional ( *Job Satisfaction Survey*) con variables sociodemográficaslaborales. Fueron aplicadas las pruebas t de Student, Mann-Whitney, Anova con posprueba de Tukey, Kruskal-Wallis o Dunn para verificar la significancia estadística de los resultados con nivel crítico de 0,05.

**Resultados:**

noventa profesionales se mostraron satisfechos con su trabajo, tres profesionales se mostraron insatisfechos y doscientos cuarenta y un profesionales mostraron ambivalencia.

**Conclusión:**

Se evidenció ambivalencia en los trabajadores de enfermería en relación a su satisfacción laboral durante el período de la pandemia de COVID-19. Los hallazgos señalan un camino para que los gestores y formuladores de políticas de salud encaucen inversiones en planes de carrera y ambientes laborales que mejoren las condiciones de los trabajadores de enfermería.

## Abstract (pt)

**Objetivo:**

avaliar a satisfação com o trabalho de profissionais de enfermagem que atuaram na assistência e na gestão, durante a pandemia de COVID-19.

**Método:**

estudo epidemiológico de corte transversal. Participaram 334 profissionais de enfermagem de um hospital de ensino. Foram calculadas as frequências absolutas e relativas das variáveis qualitativas e as médias das variáveis numéricas. Foram comparadas as médias dos resultados dos domínios da escala de satisfação profissional (Job Satisfaction Survey) com variáveis sociodemográficas-laborais. Foram aplicados os testes t de Student, Mann-Whitney, Anova seguidos de pós-teste de Tukey, Kruskal-Wallis ou Dunn, para verificar a significância estatística dos resultados com nível crítico de 0,05.

**Resultados:**

90 profissionais demonstraram-se satisfeitos com o trabalho, três profissionais demonstraram-se insatisfeitos e 241 profissionais demonstraram-se ambivalentes.

**Conclusão:**

foi evidenciada uma ambivalência dos trabalhadores de enfermagem em relação à sua satisfação com o trabalho, durante o período da pandemia de COVID-19. Os achados indicam um caminho para que gerentes e formuladores de política em saúde façam investimentos em planos de carreira e ambiente de trabalho que melhorem as condições de trabalho dos trabalhadores de enfermagem.

**Highlights:**

**(1)** Evidenced ambivalence regarding job satisfaction.

**(2)** Evidenced dissatisfaction regarding compensation, benefits, promotion and environment.

**(3)** Evidenced dissatisfaction regarding compensation, benefits, promotion and environment.

**(4)** Evidenced dissatisfaction regarding compensation, benefits, promotion and environment.

## Introduction

Healthcare systems worldwide are able to operate at full capacity for a few months, but healthcare workers do not have this power. People simply cannot be compared to fans or production machines, because they cannot be manufactured or run on an emergency basis and remain in that position for long periods. Health managers must consider health professionals as the most valuable resource when it comes to fighting the COVID-19 pandemic ^( 1)^ . 

Nursing professionals remain in hospitals experiencing precarious personal protection equipment, prolonged exposure to a large number of infected patients (increased viral load), and sudden increases in the pace of work, which added to an insufficient number of nursing workers. All these related factors increase health professionals’ risk of infection and, consequently, anxiety ^( 2)^ . 

In an analysis of the COVID-19 pandemic period, health professionals experienced a completely new situation, with difficult decisions to be made under pressure ^( 3)^ . These decisions included: allocating scarce or limited resources to equally high-need patients, balancing personal and professional demands, coping with sudden increases in workload, and dealing with absenteeism. These factors were generators of moral damage or mental health problems, such as psychological suffering resulting from the lack of actions and which violates their moral or ethical code ^( 3)^ . 

In addition, the essentiality of nursing activities stood out in such a way that, during the pandemic and situations of restricted activities and mobility, nursing professionals kept working without neglecting the responsibilities of caring for family members such as children or elderly parents.

Nursing is considered a stressful profession, i.e., subject to the set of reactions that occur in an organism when subjected to the effort of adaptation, generating physical and psychological health problems ^( 4)^ . 

Thus, the illness process of nursing professionals can be caused by the routine occurrence of situations such as fragmentation of tasks and relationships, inflexible hierarchical structure, inadequate staff sizing, high absenteeism, inadequate working conditions, overload of tasks, poor remuneration, professional dissatisfaction, and exposure to losses, suffering, and death ^( 4)^ . 

Preliminary data from a study with a total sample of 1,379 health professionals who worked in health care during the COVID-19 pandemic pointed out that the professionals reported experiencing symptoms of post-traumatic stress, depression, anxiety, insomnia, and stress ^( 5)^ . 

Although there is no single model of the composition of the determinants of job satisfaction, there have been discussions and studies on the subject since 1969. Some models consider the worker’s evaluation of his/her function, which should be translated into a positive feeling of well-being if there is satisfaction ^( 6)^ . However, some models delve into the factors that would lead to dissatisfaction, such as increased workload, private life impaired by work, remuneration, and repetitive or monotonous activities in the daily work routine ^( 7- 8)^ . 

Job satisfaction is not static. It is influenced by internal and external factors in the work environment and can be understood as a feeling of well-being with what one does, satisfaction of needs, and self-fulfillment in the work environment. Considering that job satisfaction generates pleasure and motivation, specifically in nursing, it can positively impact patient care through greater involvement of these workers, with professional and institutional commitment ^( 9)^ . 

Despite the complexity of the topic and the fact that there is no consensus regarding the definition of job satisfaction, there are several theories that address satisfaction from different perspectives. One of the classic concepts of job satisfaction is Locke’s Theory. This theory considers job satisfaction to be a function of the relationship between what individuals want from their job and what they perceive they get. Job satisfaction can be considered a pleasurable emotional state, resulting from the evaluation of work in relation to individuals’ values ^( 10)^ . 

Given the uninterrupted nursing work during the COVID-19 pandemic, the assessment of nursing professionals’ satisfaction can contribute to guiding managerial actions to promote professional satisfaction in nursing or minimize impacts on the mental health of these professionals and, consequently, promote greater safety and quality of care.

Thus, the objective of this study was to assess job satisfaction among nursing professionals who worked in nursing care and management during the COVID-19 pandemic.

## Method

### Design

This is a quantitative, correlational, cross-sectional study. The study followed the Strengthening the Reporting of Observational Studies in Epidemiology (STROBE) guidelines for observational studies, recommended by the EQUATOR network.

### Setting

The study was conducted in the nursing department of a large general teaching hospital in Campinas, in the interior of the state of São Paulo, which serves patients of the Brazilian Unified Health System (SUS). The nursing department is responsible for the technical management of nursing professionals hired through a public exam or selection process. Nurses and nursing technicians who provide direct patient care have a 30-hour weekly workload, whereas nurse managers have a 40-hour weekly workload.

### Data collection

Data collection was conducted between June and August 2021, by means of an electronic form using Google Forms. Fifteen minutes were estimated for the participants to answer the survey.

### Data collection instrument

The Job Satisfaction Survey (JSS) - Brazilian version ^( 10)^ was used. This is a public domain instrument developed in the United States in 1985 ^( 11)^ and based on the comparison between the working conditions desired by workers and the conditions experienced in their current job. 

The JSS is made up of nine assessment domains and each domain has four items assessed by a six-point Likert scale, which can range from “strongly disagree” to “strongly agree” ^( 10- 11)^ . The domains are Remuneration, Promotion, Supervision, Benefits, Rewards, Operational Procedures, Colleagues, Nature of Work, and Communication. 

Each participant was instructed to complete all items on the instrument. In the evaluation of responses to the JSS, job satisfaction can vary from low (dissatisfied) to high (satisfied). The analysis of the results for each domain considers that scores between 4 and 12 points indicate dissatisfied individuals; between 16 and 24 points, satisfied individuals, and from 13 to 15 points, individuals who are neither satisfied nor dissatisfied. Regarding the total score of the instrument, values between 36 and 108 points represent dissatisfied professionals; values between 109 and 143 indicate that the professionals are ambivalent, that is, neither satisfied nor dissatisfied with their work; and, satisfied professionals have a total score between 144 and 216 points ^( 10- 11)^ . 

### Participants

Given the limitations imposed by the pandemic for face-to-face data collection, all 1,407 nursing professionals working at the study hospital were invited to participate by e-mail. The sample was made up by convenience, considering that any nurse or nursing technician working at the institution during the data collection period could participate in the study. A total of 367 answers were obtained, but 26 participants sent duplicate answers, and only the second one was considered. Of the 341 respondents, seven did not answer the instrument completely, thus 334 participants were considered for the study, namely 196 nursing technicians and 138 nurses.

### Ethical aspects

The research project was reviewed by the research ethics committee of the university where the researchers are affiliated and, after approval through opinion number 4.421.122 9/2021, the professionals were invited to participate in the study. The professionals were informed about the study’s purpose and anonymity in data collection to become study participants, and they could only answer the JSS after reading and signing an informed consent form.

### Data processing and analysis

The data was stored in spreadsheets created in Microsoft Excel for Windows 2010 ^®^. The statistical software Statistical Analysis System, SAS version 9.4, was used for all data analyses. 

The means of the results of the domains of the Job Satisfaction Survey were compared with sociodemographic-labor variables. For comparisons involving two groups and the results of a quantitative variable, unpaired Student’s t-test or Mann-Whitney test were applied, according to data distribution ^( 12)^ . For comparisons involving more than two groups and the results of a quantitative variable, the Anova model was applied followed by Tukey’s post-test, or the non-parametric Kruskal- Wallis test followed by Dunn’s post-test, according to data distribution ^( 12)^ . The data distribution was evaluated by the Shapiro-Wilk test and a 0.05 significance level was adopted ^( 12)^ . 

## Results

Table 1 shows the sociodemographic variables: sex, marital status, level of education, professional category, and working shift of the study participants who made up the final sample. 

**Table 1 -:** Sociodemographic variables, sex, marital status, level of education, and working shift by professional category (n *=334). Campinas, SP, Brazil, 2021

	Nursing technician	Nurse (care)	Nurse (manager)	n *	%
**Sex**				334	100
Female	170	92	26	288	86.23
Male	26	13	7	46	13.77
**Marital status**				334	100%
With a partner	128	75	24	227	67.96
Without a partner	68	30	9	107	32.04
**Level of education**				334	100
Complete technical education	108			108	32.34
Incomplete higher education		27		27	8.08
Complete higher education			161	161	48.20
Postgraduate degree				38	11.38
**Working shift**				334	100
Morning	48	29	2	79	23.65
Afternoon	49	32	1	82	24.55
Night	87	38	8	133	39.82
Business hours	12	6	22	40	11.98

* n = Number of respondents

Regarding total score and indication of job satisfaction, 3 professionals were dissatisfied (score between 96-108), 241 were ambivalent about job satisfaction (score between 109-143) and 90 professionals were satisfied with their jobs (score between 144-172).

The results of the comparison between the means of the JSS domains by sex and marital status did not show statistically significant differences (p>0.05). Table 2 shows the comparison between the mean scores of the domains and the total JSS by education. 

**Table 2 -:** Comparison between the means of the domains and the total score in the Job Satisfaction Survey by education (n *=334). Campinas, SP, Brazil, 2021

Variable	Complete technical education	Incomplete higher education	Complete higher education	Post-graduate degree
Remuneration ^†^	9.57	9.7	9.38	8.97
Benefits ^†^	9.59	9.63	9.69	10.42
Promotion ^†^	6.11	6.15	6.07	6.87
Colleagues ^[Table-fn t2f3b]‡^	17.35	17.37	16.66	15.24
Rewards ^†^	11.50	11.96	11.39	11.39
Nature of the work ^†^	15.31	15.70	14.76	14.03
Supervision ^†^	19.42	18.89	18.80	19.16
Operational conditions ^[Table-fn t2f3b]‡^	9.67	9.93	8.44	6.97
Communication ^†^	14.04	14.74	13.82	12.16
Total ^†^	112.36	114.07	108.83	104.92

* n = Number of respondents;

† p-value>0.05, obtained using Kruskal-Wallis test;

‡ p-value<0.05, obtained using Kruskal-Wallis test

In Dunn’s post-test, it was verified that the differences in the means are statistically significant in the domains: Colleagues with education between complete technical education and post-graduate degree (p-value=0.012); and Operational Conditions between complete technical education and complete higher education (p-value=0.022), between complete technical education and post-graduate degree (p-value<0.001) and between incomplete higher education and post-graduate degree (p-value<0.001).

Table 3 shows the comparison between the means of the domains and the total JSS by position held. 

**Table 3 -:** Comparison between the means of the domains and the total score in the Job Satisfaction Survey by position (n *=334). Campinas, SP, Brazil, 2021

Variable	Nursing technician	Nurse (care)	Nurse (manager)
Remuneration ^†^	9.48	8.77	11.12
Benefits ^‡^	9.64	9.73	10.29
Promotion ^†^	5.73	6.07	9.12
Colleagues ^‡^	17.12	16.41	15.94
Rewards ^‡^	11.28	11.36	12.94
Nature of the work ^‡^	15.19	14.63	14.41
Supervision ^‡^	19.36	18.23	19.79
Operational conditions ^†^	9.45	8.24	6.68
Communication ^‡^	14.16	13.25	13.21
Total ^‡^	111.16	106.69	113.39

* n = Number of respondents

† p-value<0.05, obtained using Kruskal-Wallis test;

‡ p-value>0.05, obtained using the ANOVA test

Dunn’s post-test showed a statistically significant difference between the means of nursing technicians and nurse managers in the Remuneration (p-value=0.030), Promotion and Operational Conditions (p-value<0.001) domains; and between nurses involved in care and nurse managers in the Remuneration (p-value=0.003), Promotion (p-value<0.001) and Operational Conditions (p-value=0.028) domains.

Table 4 shows the comparison between the means of the domains and the total score in the JSS by working shift. 

**Table 4 -:** Comparison between the means of the domains and the total score in the Job Satisfaction Survey by working shift (n *=334). Campinas, SP, Brazil, 2021

Variable	Morning	Afternoon	Night	Business hours
Remuneration ^†^	8.80	10.25	9.25	9.50
Benefits ^‡^	9.11	10.93	9.45	9.50
Promotion ^‡^	5.60	6.59	5.90	7.40
Colleagues ^‡^	15.83	17.33	17.37	15.55
Rewards ^‡^	10.15	13.20	11.19	11.53
Nature of the work ^‡^	14.49	15.57	15.10	13.93
Supervision ^‡^	18.68	20.10	18.63	19.00
Operational conditions ^‡^	8.33	9.64	9.08	6.95
Comunicação ^‡^	12.83	14.98	14.00	12.46
Total ^‡^	103.83	118.38	109.71	105.66

* n = Number of respondents

† p-value<0.05, obtained using Kruskal-Wallis test;

‡ p-value>0.05, obtained using the ANOVA test

The Tukey post-test showed a statistically significant difference between morning and night shifts (p-value = 0.001) and between night and business hours (p-value = 0.044) for the JSS total score.

Dunn’s post-test showed a statistically significant difference between afternoon and night mean scores in the Benefits (p-value=0.050), Rewards (p-value=0.032), and Supervision (p-value=0.033) domains. There was a statistically significant difference between morning and night means only in the Colleagues domain (p-value=0.014). When comparing the means between morning and afternoon shifts, there was a statistically significant difference in the Benefits (p-value=0.019), Colleagues (p-value=0.044), Rewards (p-value=0.032), Operational Conditions (p-value=0.044) and Communication (p-value=0.013) domains.

## Discussion

This study sought to analyze the job satisfaction of nursing professionals from a tertiary-level university hospital, highlighting that the participating professionals never had the possibility of remote work during the COVID-19 pandemic.

Considering the JSS total score, there was a predominance of ambivalent professionals regarding their job satisfaction in the sample studied, which was similar to the results of another national study ^( 13)^ . 

Most participants were female and married, evidencing that in the sample, Nursing is essentially female, and culturally considered a woman’s attribution in relation to health care ^( 14- 17)^ . Among frontline health professionals, women are a risk group for mental suffering because they perform tasks that require a high level of contact with patients, which increases the fear of contagion ^( 14- 15)^ . In addition, women are culturally expected to make sacrifices for the benefit of others, carrying out household chores and caring for children, the elderly, or sick people, which contributes to their work overload ^( 14- 17)^ . However, in the present study, job satisfaction did not differ between sexes, nor even between professionals with or without a partner. In all participant groups, values consistent with job satisfaction by the JSS were only found in the Supervision and Colleagues domains. 

It was noted that the study population seeks knowledge/specialization. Despite having 58% of nursing technicians working in this function, only 32% of the respondents said they had only a complete technical education, whereas another 48% of nursing professionals had a complete higher education and 11% had a post- graduate degree, showing an interest in growing in the profession and valuing nursing. Although among all levels of education only the Colleagues and Supervision domains have shown job satisfaction, it is important to note that with regard to the Colleagues and Operational Conditions domains, professionals with post-graduate degrees had lower means compared to professionals with complete technical education and incomplete higher education.

Considering the study population, 58% of the respondents worked as nursing technicians and 48% of the respondents reported having a complete higher education, i.e., there is a relative portion of nursing technicians who aspire an opportunity to work as nurses, as well as a salary increase, and this may have contributed for the professionals with complete higher education to feel dissatisfied in relation to their job. In this sense, given the fact that a contingent of nursing technicians have schooling qualifications similar to those of their immediate superiors (Table 3) and remain in mid-level positions while awaiting the opportunity of a public exam to be hired as nurses, it can be considered a risk or a triggering condition for conflicts in power relations and, consequently, promote mental health problems among these professionals ^( 18- 21)^ . 

Nursing is characterized by categories and its historical evolution demonstrates a growing technical and social division of labor ^( 18- 25)^ . Nursing is characterized by categories and its historical evolution demonstrates a growing division of labor - technical and social ^( 18- 25)^ . Nurses are responsible for the administration, supervision, and control of care and management activities (intellectual activities), while direct patient care activities (manual activities) are developed by other nursing agents. The permanent class struggles reflect a social and technical division of nursing work, which has been perpetuated in order to meet political, social, and economic interests ^( 18- 25)^ . This culture may justify the results of this study, with the ambivalence of managers and nursing technicians and the dissatisfaction of nurses working in care, especially in the field of operational working conditions. 

Nursing work is focused on care management and nursing service management, which is characterized by involving people and material management activities to reach organizational goals. Care management is an inherent attribution of the nurse’s professional practice. The management of nursing services is the responsibility of nurse managers or heads of units ^( 17- 18, 21- 23)^ . The two spheres of nursing work are interconnected in the search for excellence in patient care, and the perseverance of professionals has been associated with job satisfaction ^( 17, 22, 24)^ . 

Although no studies were found aiming to assess job satisfaction in times of pandemics, a national study indicated that nursing technicians and aides showed a significant association with suffering at work, in addition to low pleasure, which was attributed to the demands of the nature of their work, as well as low social support ^( 15)^ . 

In this sense, it is important to emphasize that not only the physical and organizational conditions of the nursing work environment influence the best practice of nursing professionals. Factors such as the size of the institution or health service, management model, professional hierarchies, organizational culture, infrastructure, and human and financial resources for the performance of care can enhance or limit nursing practice ^( 17- 18, 22)^ . Thus, work environments that have these aspects strengthened bring greater professional satisfaction to nursing, and consequently, improve the quality of nursing care in the hospital environment ^( 20, 22- 23)^ . 

The Colleagues and Supervision domains, considered positive in the study, involve the relationship among team members and have relevant representativeness regarding pleasure at work and quality of life of workers and may impact quality of care and patient safety ^( 14- 15, 21, 24, 26- 28)^ . Given the positive evaluation in these domains that involve the relationship among nursing team members and the relationship with the immediate superiors, the findings may represent the sense of belonging of professionals in the work team, with constructive experiences and the possibility of mutual support in facing the difficulties inherent to the pandemic period ^( 14, 24- 28)^ . 

Operational Conditions, a domain that presented averages consistent with job dissatisfaction, should be compared longitudinally because they may have been worsened in the waves of the COVID-19 pandemic. Due to the abrupt increase in work demand, the domain may have captured workers’ perceptions of task fragmentation, insufficient staffing, absenteeism, turnover, and exposure to suffering and death ^( 1, 7, 15- 16)^ . 

A survey of healthcare professionals in Portugal revealed satisfaction, more specifically the relationship between job characteristics and job satisfaction on turnover, which reflects directly on organizational commitment. It concluded that the higher the satisfaction, the higher the commitment, the lower the impact on turnover and absenteeism, and the better the perception of customer satisfaction specifically in healthcare organizations ^( 23)^ . 

The leadership practice and organizational commitment evaluated in the Supervision and Operational Conditions domains are indicators of potential or achieved improvements in the work process and consequently drivers of job satisfaction among nursing workers ^( 7, 16, 19, 21, 26- 28)^ . 

In this direction, the analysis of the Remuneration, Promotion, Benefits, Rewards, Operational Procedures, Nature of Work, and Communication domains indicate managerial opportunities to prioritize actions for staff motivation. While job satisfaction can be a pleasure-generating force, albeit a subjective and personal state, motivation is also considered a multidimensional construct, whose expression is the result of a comparison between what was obtained and what one wanted to obtain from work ^( 21- 22, 28)^ . 

In the comparative analysis in relation to working shifts, the best levels of satisfaction of the professionals assigned to afternoon shifts stand out. With the limitation of not having compared the number of jobs between shifts, it is important to highlight that the hospital where the data was collected has a 30-hour workweek and a higher starting salary than other hospitals in the region.

It is believed that satisfaction and work context are not limited to the physical or social space in which the activities are developed, but also include interpersonal relationships; the perception of workers about how important their work is, with recognition by society; the service dynamics, with the availability of minutes during the shift for discussions and reflections; the stimulus to experience humor, altruism and self-observation; and organizational support with the creation of physical spaces for workers to rest ^( 7, 16, 19, 26- 28)^ . 

From this perspective, the JSS author ^( 11)^ highlights that job satisfaction is a feeling experienced by professionals about their work and reflects how much people like or dislike their work, denoting a constellation of attitudes related to various aspects of the occupational environment ^( 11)^ . In this sense, the experience of pleasure may result from the relationship between professional achievement and freedom of expression, involving aspects such as well-being, satisfaction, motivation, recognition and pride for the activities performed and opportunities to express opinions to colleagues and supervisors ^( 15- 16, 19, 27- 28)^ . 

During the work in the pandemic period, the impossibility of holding conversation circles associated with the negative experiences of nursing workers, such as emotional exhaustion, stress, dissatisfaction, overload, frustration, insecurity, feelings of devaluation, indignation, uselessness, disqualification, injustice, discrimination, and lack of recognition ^( 7, 15, 19, 25- 27)^ , may have interfered in the completion of the instrument, including discouraging professionals to participate. 

The limitations of this study are the lack of randomization of the sample and its cross-sectional design, which does not allow for establishing a causal link, but rather indicates an association between variables. In addition, no studies that used the JSS during the COVID-19 pandemic were found, which would allow comparability with the findings of this study.

This study indicated the importance of recognizing and rewarding the nursing professionals who worked uninterruptedly during the COVID-19 pandemic with actions, policies, and strategies that go beyond the transience of clapping hands and contemplate reviews of work processes, career plans, possibilities of rewards, and a careful look at working conditions with the acceptance of fears and possibilities of continued reflections.

The study also revealed the professionals’ recognition in relation to their supervision and coexistence with co-workers, in such a way that these two were the only dimensions of job satisfaction considered positive among all the actors of care in the study institution: nurse managers, nurses involved in care, and nursing technicians.

## Conclusion

In the present study, ambivalence among the nursing workers was evidenced regarding their job satisfaction during the period of the COVID-19 pandemic.

Concerning education, there was no significant difference regarding job satisfaction as a whole; however, in the Colleagues domain, satisfaction was higher among professionals with technical education; and, in the Supervision domain, professionals with postgraduate degrees showed higher satisfaction compared to professionals with technical or higher education.

In general, professional dissatisfaction was registered by the participants regarding Remuneration, Benefits, Promotion, and Operational Conditions; ambivalence or neutrality regarding job satisfaction was related to the Communication and Nature of Work domains; and professional satisfaction was evidenced by the participants regarding Colleagues and Supervision.

Nurse managers showed better job satisfaction regarding remuneration, promotion, and operational conditions than nurses involved in care and nursing technicians.

The findings indicate a path for managers and health policymakers to invest in career plans and the work environment to improve the conditions for nursing workers to act with safety and dignity in patient care.
